# Panitumumab and irinotecan versus irinotecan alone for patients with *KRAS* wild-type, fluorouracil-resistant advanced colorectal cancer (PICCOLO): a prospectively stratified randomised trial

**DOI:** 10.1016/S1470-2045(13)70163-3

**Published:** 2013-07

**Authors:** Matthew T Seymour, Sarah R Brown, Gary Middleton, Timothy Maughan, Susan Richman, Stephen Gwyther, Catherine Lowe, Jennifer F Seligmann, Jonathan Wadsley, Nick Maisey, Ian Chau, Mark Hill, Lesley Dawson, Stephen Falk, Ann O'Callaghan, Kim Benstead, Philip Chambers, Alfred Oliver, Helen Marshall, Vicky Napp, Phil Quirke

**Affiliations:** a Cancer Medicine and Pathology, University of Leeds, Leeds, UK; b Clinical Trials Research Unit, University of Leeds, Leeds, UK; c School of Cancer Sciences, University of Birmingham, Edgbaston, UK; d University of Oxford, Oxford, UK; e East Surrey Hospital, Surrey, UK; f Weston Park Hospital, Sheffield, UK; g Guy's and St Thomas's Hospitals, London, UK; h Royal Marsden Hospital and NIHR Biomedical Research Centre, Sutton, UK; i Kent Oncology Centre, Maidstone, UK; j Edinburgh Cancer Centre, Western General, Edinburgh, UK; k Bristol Haematology and Oncology Centre, Bristol, UK; l Queen Alexandra Hospital, Portsmouth, UK; m Cheltenham General Hospital, Cheltenham, UK; n NCRN Consumer Liaison Group, Leeds, UK

## Abstract

**Background:**

Therapeutic antibodies targeting EGFR have activity in advanced colorectal cancer, but results from clinical trials are inconsistent and the population in which most benefit is derived is uncertain. Our aim was to assess the addition of panitumumab to irinotecan in pretreated advanced colorectal cancer.

**Methods:**

In this open-label, randomised trial, we enrolled patients who had advanced colorectal cancer progressing after fluoropyrimidine treatment with or without oxaliplatin from 60 centres in the UK. From December, 2006 until June, 2008, molecularly unselected patients were recruited to a three-arm design including irinotecan (control), irinotecan plus ciclosporin, and irinotecan plus panitumumab (IrPan) groups. From June 10, 2008, in response to new data, the trial was amended to a prospectively stratified design, restricting panitumumab randomisation to patients with *KRAS* wild-type tumours; the results of the comparison between the irinotcan and IrPan groups are reported here. We used a computer-generated randomisation sequence (stratified by previous EGFR targeted therapy and then minimised by centre, WHO performance status, previous oxaliplatin, previous bevacizumab, previous dose modifications, and best previous response) to randomly allocate patients to either irinotecan or IrPan. Patients in both groups received 350 mg/m^2^ intravenous irinotecan every 3 weeks (300 mg/m^2^ if aged ≥70 years or a performance status of 2); patients in the IrPan group also received intravenous panitumumab 9 mg/kg every 3 weeks. The primary endpoint was overall survival in *KRAS* wild-type patients who had not received previous EGFR targeted therapy, analysed by intention to treat. Tumour DNA was pyrosequenced for *KRAS*_c.146_, *BRAF, NRAS*, and *PIK3CA* mutations, and predefined molecular subgroups were analysed for interaction with the effect of panitumumab. This study is registered, number ISRCTN93248876.

**Results:**

Between Dec 4, 2006, and Aug 31, 2010, 1198 patients were enrolled, of whom 460 were included in the primary population of patients with *KRAS*_c.12–13,61_ wild-type tumours and no previous EGFR targeted therapy. 230 patients were randomly allocated to irinotecan and 230 to IrPan. There was no difference in overall survival between groups (HR 1·01, 95% CI 0·83–1·23; p=0·91), but individuals in the IrPan group had longer progression-free survival (0·78, 0·64–0·95; p=0·015) and a greater number of responses (79 [34%] patients *vs* 27 [12%]; p<0·0001) than did individuals in the irinotecan group. Grade 3 or worse diarrhoea (64 [29%] of 219 patients *vs* 39 [18%] of 218 patients), skin toxicity (41 [19%] *vs* none), lethargy (45 [21]% *vs* 24 [11%]), infection (42 [19%] *vs* 22 [10%]) and haematological toxicity (48 [22%] *vs* 27 [12%]) were reported more commonly in the IrPan group than in the irinotecan group. We recorded five treatment-related deaths, two in the IrPan group and three in the irinotecan group.

**Interpretation:**

Adding panitumumab to irinotecan did not improve the overall survival of patients with wild-type *KRAS* tumours. Further refinement of molecular selection is needed for substantial benefits to be derived from EGFR targeting agents.

**Funding:**

Cancer Research UK, Amgen Inc.

## Introduction

In 2003, therapeutic antibodies targeting EGFR entered phase 3 trials in advanced colorectal cancer. In December, 2006, the UK Colorectal Clinical Studies Group launched a randomised trial in fluorouracil-resistant advanced colorectal cancer, called the Panitumumab, Irinotecan, and Ciclosporin in COLOrectal cancer (PICCOLO) trial. We selected patients using conventional clinicopathological criteria and allocated them randomly in equal distributions to one of three groups: irinotecan alone, irinotecan plus ciclosporin, or irinotecan plus panitumumab (IrPan).

In April 2008, *KRAS* mutation was reported to be a negative predictive biomarker for EGFR targeted therapy—retrospective analysis of a randomised trial^1^ of panitumumab versus supportive care showed that panitumumab benefit was confined to patients with tumours wild-type at *KRAS* codons 12–13 (p<0·0001). Two months later, retrospective analysis of two further randomised trials^2^, ^3^ showed similar results for cetuximab. By that time, we had recruited 494 of the planned 1269 patients to PICCOLO. The Trial Management Group (including patients representatives) and an independent data monitoring and ethics committee agreed that continued randomisation of patients with *KRAS* mutations to panitumumab would not be beneficial to the patients nor would it provide useful data. The aim of the trial was therefore amended: evaluation of panitumumab would now focus on patients with *KRAS* wild-type tumours, with quantification of treatment benefit and evaluation of further biomarkers in this selected population, rather than in an unselected population. On June 10, 2008, 1 week after announcement of the cetuximab data, a safety amendment was introduced to exclude patients with *KRAS*-mutated tumours from randomisation to the IrPan group; within 3 months PICCOLO was reopened as a prospectively stratified trial: patients with *KRAS* wild-type tumours were randomly allocated to irinotecan or IrPan while those with *KRAS* mutations (or unknown *KRAS* status) were randomly allocated to irinotecan or irinotecan plus ciclosporin. We present here the final results of the irinotecan versus IrPan comparison for patients with *KRAS* wild-type tumours who had not received previous anti-EGFR therapy; findings from the irinotecan versus irinotecan plus ciclosporin comparison will be reported elsewhere.^4^

## Methods

### Study design and patients

PICCOLO was a multicentre, randomised controlled trial in chemoresistant advanced colorectal cancer. Recruitment of molecularly unselected patients started on Dec 4, 2006; panitumumab randomisation was restricted to known *KRAS*-wild type patients from June 10, 2008; it was then relaunched with full prospective molecular stratification from Aug 31, 2008, and closed to recruitment on Aug 31, 2010.

We recruited patients from 60 centres in the UK. Eligible patients were aged 18 years or older, had histologically confirmed colorectal cancer, inoperable advanced disease, and had progressed during or after fluoropyrimidine-containing chemotherapy. Patients could have received any previous drugs except for irinotecan. Other eligibility criteria were as follows: Response Evaluation Criteria In Solid Tumors (RECIST) measurable disease;^5^ WHO performance status 0–2; haemoglobin concentration of 100 g/L or greater; white blood cell count of greater than or equal to 3·0×10^9^ cells per L; a platelet count of greater than or equal to 100×10^9^ per L; estimated glomerular filtration rate of greater than or equal to 50 mL min^−1^; bilirubin concentration less than or equal to 25 μmol/L; and alkaline phosphatase concentrations of five times the upper limit of normal or lower and aminotransferase concentrations of 2·5 times upper limit of normal or lower.

Nationwide ethical approval was obtained. Before enrolment, patients provided written consent to participate, and for the molecular studies.

### Randomisation and masking

From Dec 4, 2006, to June 9, 2008, patients were allocated equally to irinotecan alone, irinotecan plus ciclosporin, or IrPan. Randomisation was done with an automated telephonic system at the Clinical Trials Research Unit, University of Leeds, UK, using a computer-generated minimisation algorithm including a random element, first stratified by previous treatment with EGFR monoclonal antibodies, then minimised within each of the following strata: centre, WHO performance status, previous oxaliplatin, previous bevacizumab, previous dose modifications, and best previous response.

On June 10, 2008, a temporary safety measure was applied that restricted the allocation of patients with unknown or mutated *KRAS* status to the irinotecan or irinotecan plus ciclosporin groups only. Regulatory and ethical approval of a fully amended, molecularly stratified protocol was obtained on Aug 4, 2008. Under the new protocol, patients were pre-registered (either when PICCOLO therapy was indicated or pre-emptively during first-line therapy) and stored resection or biopsy tumour material was retrieved and tested for *KRAS*_c.12,13,61_. To reduce the possibility of patients in the control group (irinotecan only) being discontinued from treatment prematurely, and after consultation with the patient representative on the Trial Management Group, patients and their clinicians were not routinely made aware of patients' *KRAS* status, but the information was available on request. Randomisation occurred immediately before starting treatment.

In the amended protocol, randomisation was stratified by *KRAS* status: patients with *KRAS* wild-type tumours were randomised in a one-to-one ratio to irinotecan or IrPan. If *KRAS* was mutated or unknown, randomisation was one-to-one to irinotecan or irinotecan plus ciclosporin.

Randomisation in each comparison was via minimisation, incorporating a random element adjusting for the same minimisation factors under the original protocol. In the irinotecan versus IrPan comparison, patients were first stratified by previous EGFR targeted therapy, with minimisation done separately within each stratum. This was an open-label trial, so patients and clinicians were not masked to treatment allocation.

### Procedures

The full protocol is available online. Briefly, all patients received an intravenous infusion of irinotecan 350 mg/m^2^ every 3 weeks (300 mg/m^2^ if aged >70 years or if they had a performance status of 2); patients in the IrPan group also received an intravenous infusion of panitumumab 9 mg/kg every 3 weeks (a schedule based on previous pharmacokinetic and pharmacodynamic data^6^). We followed detailed schemes for the management of toxicity, including treatment delays and dose reductions (full details given in the protocol). Briefly, a 1-week delay was given for unresolved non-haematological toxicities of grade 2 or higher; patients who had toxicities of grade 3 or higher, or a toxicity requiring two dose delays, had a 20% dose reduction. Treatment continued until disease progression or unacceptable toxicity. After 12 weeks (four cycles) patients with stable or responding disease could, at the clinicians' discretion, be offered a planned break from irinotecan of up to two cycles; patients on IrPan continued panitumumab alone during irinotecan breaks. There was no within-protocol crossover, but post-trial treatment was not restricted.

RECIST^5^ response was assessed every 12 weeks with CT scans, scored locally, and quality-assured by central review in more than a third of patients. Toxicity was scored using NCI Common Terminology Criteria for Adverse Events (version 3.0). Quality of life was assessed at baseline, week 12, and week 24 with EORTC QLQ-C30,^7^ EQ-5D,^8^ and Dermatology Life Quality Index.^9^

Laboratory methods are described in the appendix and elsewhere.^10^ Quality assured DNA pyrosequencing was done at the Cancer Research UK Genomics Facility, University of Leeds, UK. *KRAS*_c.12,13,61_ was assessed first; *KRAS*_c.12,13,61_ wild-type tumours were then assessed at nine further codons provided sufficient DNA was available: *BRAF*_c.600_, *NRAS*_c.12,13,61_, *KRAS*_c.146_, *PIK3CA*_c.542,545-6_ (exon 9), and *PIK3CA*_c.1047_ (exon 20).

### Statistical analysis

Under the original design, we aimed to detect a 25% reduction in hazard rate (80% power; 5% significance level; two-sided log-rank test) for the primary endpoint, overall survival, with the addition of panitumumab to irinotecan. Anticipated median overall survival with irinotecan was 9 months,^11^ with a targeted improvement to 12 months with the addition of panitumumab, resulting in a sample size of 720 patients and at least 380 deaths.

In the amended design, we anticipated an increased treatment benefit with IrPan in the refined primary population of *KRAS*_c.12,13,61_ wild-type patients not pre-treated with EGFR monoclonal antibodies. We have previously assessed *KRAS* as a prognostic and predictive marker in patients treated with cytotoxic chemotherapy alone,^10^ and on the basis of these data, we made no change to the predicted overall survival of 9 months for *KRAS* wild-type patients in the irinotecan alone group. However, in the new design we aimed to detect a 30% reduction in hazard rate, corresponding to a median overall survival of 12·9 months with the addition of panitumumab. Target accrual was 466 patients in the primary population, with the analysis planned after at least 246 deaths had occurred. An interim analysis was planned to address inferiority or superiority of irinotecan plus panitumumab compared with irinotecan alone, with a stringent p value of 0·001, therefore no adjustment was required in the final significance level.^12^ Secondary endpoints included progression-free survival (PFS), the proportion of patients who achieved a RECIST response, quality of life, and toxicity. Post-hoc statistical comparisons were made between the rates of grade 3 or higher events in the two groups, using univariate χ^2^ tests (or Fisher's exact test for five or fewer events) at the 5% significance level. This analysis did not account for multiple testing and its findings should be interpreted with caution.

We had two predefined exploratory populations: patients with *KRAS*_c.12,13,61_-mutated tumours randomised to irinotecan versus IrPan before the protocol modification; and patients previously treated with an anti-EGFR monoclonal antibody.

Additional analyses were later planned, before final analysis, to investigate any interaction between *BRAF*_c.600_, *NRAS*_c.12,13,61_, *KRAS*_c.146_, or *PIK3CA* status and the effect of panitumumab. In planning these analyses, molecular subgroups were predefined to determine treatment interaction with mutation status, with the pre-existing hypothesis that *KRAS*_c.12,13,61_ wild-type patients with a mutation at one of the other loci would have less benefit from panitumumab than would patients with no mutations. Patients were grouped as having any mutation (a mutation at any other one of the assessed loci) or as all wild-type (no mutations at the loci tested). In the analysis, missing data for an individual gene was imputed as wild-type, but we did a sensitivity analysis in which only patients confirmed to be wild-type at all 12 loci were classed as all wild-type. We did a second sensitivity analysis excluding *PIK3CA* mutation from the analysis.

For individual rare mutations occurring in less than 10% of patients, PICCOLO provides only minimal power (about 10%) to detect clinically significant treatment effects (eg, reduction in hazard rate of 30%). These analyses are therefore exploratory in nature and should not be over-interpreted. Cox's proportional hazards modelling, adjusting for minimisation factors, was pre-specified for overall survival and PFS. Statistical testing was post hoc for response rate and toxicity.

Primary analysis of all endpoints was scheduled after 246 deaths, as per the amended trial design. On recommendation from the data monitoring and ethics committee, we also planned a final updated analysis of overall survival when at least 2 years had passed since all patients were allocated to treatment. We report here the primary event-driven overall survival analysis in the primary population. We also report the secondary endpoints and final analysis of overall survival, in the primary population, its planned molecular subgroups, and in the exploratory population of patients with mutations at *KRAS*_c.12,13,61_. Results in patients previously treated with an anti-EGFR monoclonal antibody will be reported elsewhere, as will results for the comparison of irinotecan versus irinotecan plus ciclosporin. We used SAS (version 9.2) for all statistical analyses.

This study is registered as an International Standard Randomised Controlled Trial, number ISRCTN93248876.

### Role of the funding source

Cancer Research UK provided independent peer review and feedback on the original and revised protocols, but had no other involvement in the trial. Amgen Inc provided panitumumab and an educational grant, but had no involvement in the design, conduct, analysis, interpretation, or production of the report. The corresponding author had full access to the data and had full responsibility for the decision to submit for publication.

## Results

**Figure 1:**
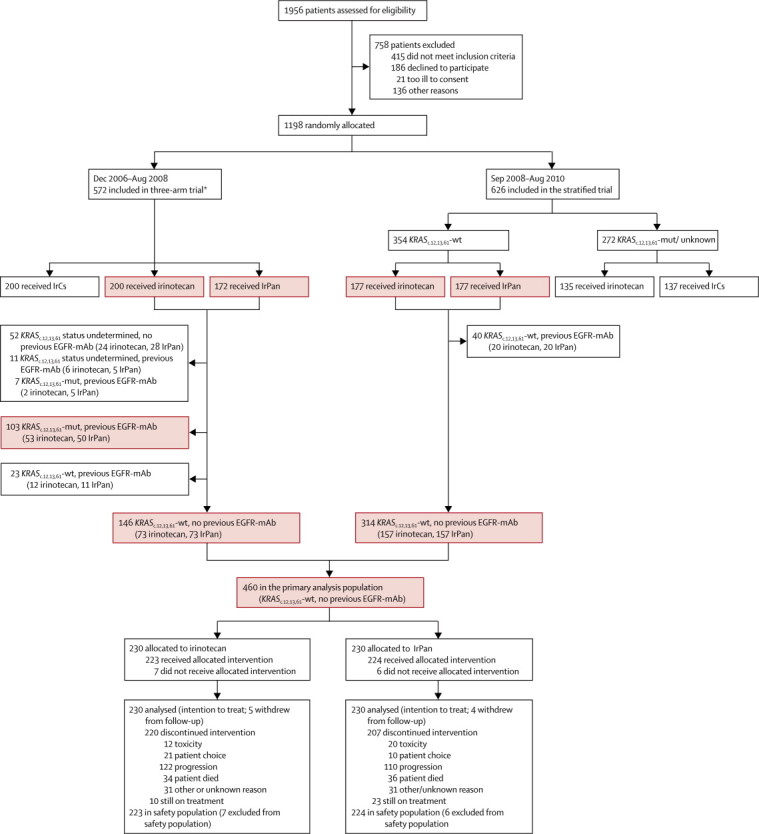
Trial profile *Between June, 2008, and August, 2008, a temporary safety measure was implemented to exclude patients with unknown or mutated *KRAS*_c.12,13,61_ status from randomisation to IrPan. 78 patients randomised during this period. 30 patients were randomised to irinotecan under the irinotecan *vs* IrCs comparison only during this time, and are not included in the summaries of patients forming the irinotecan *vs* IrPan comparison. IrCs=irinotecan plus ciclosporin. IrPan=irinotecan plus panitumumab. mAb=monoclonal antibody.

**Figure 2:**
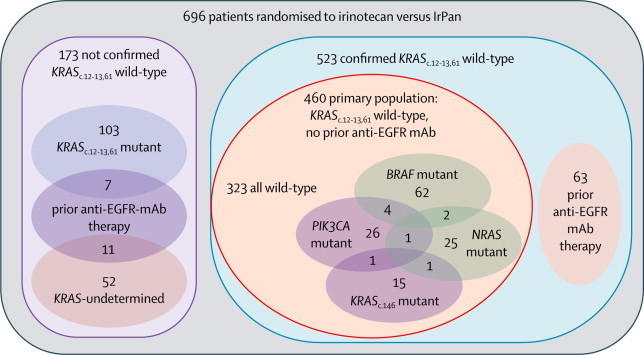
Molecular characterisation IrPan=irinotecan plus panitumumab. mAb=monclonal antibody.

Starting in December, 2006, 1198 patients were recruited to the PICCOLO trial: 494 to the initial three-arm design (Dec 4, 2006–June 9, 2008), 78 to the temporary safety protocol excluding patients with mutated or unknown *KRAS* status from the IrPan group (June 10–Aug 31, 2008) and 626 to the fully prospectively stratified design (Sept 1, 2008–Aug 31, 2010). In all, 460 patients with *KRAS* wild-type tumours who had not previously received EGFR therapy were randomly allocated to irinotecan (230 patients) or IrPan (230 patients), and these form our primary population for this report (Figure 1, Figure 2; appendix). Baseline characteristics were much the same between the two groups—most had received oxaliplatin in addition to a fluoropyrimidine and very few had received bevacizumab (table 1). Most tumour samples received for testing were from patients' primary tumour; around 5% were from metastases.

**Table 1:** Baseline characteristics

		**Irinotecan group (n=230)**	**IrPan group (n=230)**
Sex			
	Male	158 (69%)	160 (70%)
	Female	72 (31%)	70 (30%)
Age (years)		63 (56–69)	64 (57–70)
WHO performance status			
	0–1	217 (94%)	217 (94%)
	2	13 (6%)	13 (6%)
Primary tumour resected			
	Yes	179 (78%)	159 (69%)
	No	51 (22%)	71 (31%)
Previous adjuvant therapy			
	Yes	136 (59%)	134 (58%)
	No	94 (41%)	96 (42%)
Primary disease site			
	Right colon	73 (32%)	61 (27%)
	Left colon	67 (29%)	83 (36%)
	Rectum	82 (36%)	80 (35%)
	Unclear	8 (3%)	6 (3%)
Sites of disease			
	Liver	175 (76%)	166 (72%)
	Lung	115 (50%)	125 (54%)
	Mesentery or peritoneal	52 (23%)	47 (20%)
	Abdominal lymph nodes	62 (27%)	53 (23%)
	Other lymph nodes	31 (13%)	22 (10%)
	Bone	13 (6%)	12 (5%)
	Other	52 (23%)	54 (24%)
Previous bevacizumab			
	Yes	4 (2%)	5 (2%)
	No	226 (98%)	225 (98%)
Previous oxaliplatin			
	Yes	219 (95%)	217 (94%)
	No	11 (5%)	13 (6%)
Previous best response			
	Response or stable disease	150 (65%)	148 (64%)
	Progressive disease	55 (24%)	60 (26%)
	Unknown	25 (11%)	22 (10%)
*KRAS*_c.12,13,61_			
	Mutant	0	0
	Wild-type	230 (100%)	230 (100%)
	Undetermined	0	0
*BRAF*_V600E_			
	Mutant	31 (13%)	37 (16%)
	Wild-type	188 (82%)	183 (80%)
	Undetermined	11 (5%)	10 (4%)
*NRAS*_c.12,13,61_			
	Mutant	10 (4%)	19 (8%)
	Wild-type	204 (89%)	195 (85%)
	Undetermined	16 (7%)	16 (7%)
*KRAS*_c.146_			
	Mutant	8 (3%)	9 (4%)
	Wild-type	193 (84%)	190 (83%)
	Undetermined	29 (13%)	31 (13%)
*PIK3CA*_exon 9/20_			
	Mutant	21 (9%)	11 (5%)
	Wild-type	176 (77%)	171 (74%)
	Undetermined	33 (14%)	48 (21%)
No mutations detected		163 (71%)	160 (70%)
Any mutation detected		67 (29%)	70 (30%)

Data are n (%) or median (IQR). IrPan=irinotecan plus panitumumab.

Patients in both groups received a median of four treatment cycles (mean 5·9; range 0–28). 13 (6%) patients in the irinotecan group and one (<0·5%) patient in the IrPan group received an anti-EGFR monoclonal antibody as salvage therapy within 3 months of finishing trial treatment.

The primary analysis of overall survival was triggered after 246 deaths, although when the database was locked for analysis, 312 (68%) of 460 patients had died. The analysis was presented in full at an international conference in 2011:^13^ in brief, median overall survival was 10·5 months (95% CI 9·5–12·4) in the irinotecan group and 10·4 months (8·7–12·2) in the IrPan group (hazard ratio [HR] 0·91, 95% CI 0·73–1·14; p=0·44). Thus, PICCOLO did not meet its primary objective of showing improved overall survival in the primary analysis population.

**Figure 3:**
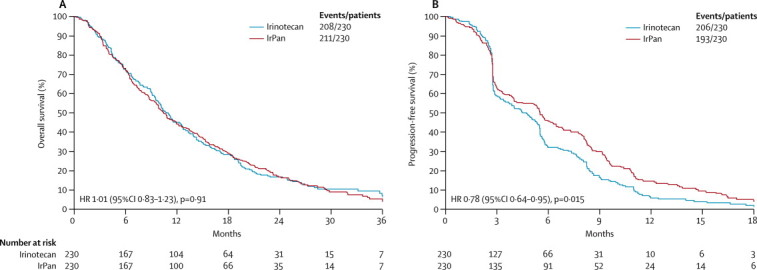
Kaplan-Meier curves of (A) overall survival and (B) progression-free survival, at final analysis IrPan=irinotecan plus panitumumab.

All further overall survival analyses presented in this report use final survival data. At final analysis, 419 (91%) of 460 patients in the primary population had died, and median follow-up of those patients still alive (n=41) was 25·4 months (IQR 22·5–30·8). At final analysis, we recorded no difference in overall survival between the groups: median survival was 10·9 months (95% CI 9·5–12·5) in the irinotecan group and 10·4 months (8·9–12·2) in the IrPan group (HR 1·01, 95% CI 0·83–1·23; p=0·91; figure 3).

Secondary endpoints were analysed after data cleaning at the time of the primary event-driven overall survival analysis. PFS was longer in the IrPan group than in the irinotecan group (HR 0·78, 95% CI 0·64–0·95; p=0·015; figure 3). More patients had a RECIST-defined response in the IrPan group than in the irinotecan group (table 2), with a multivariate odds ratio of 4·12 (95% CI 2·52–6·76; p<0·0001).

**Table 2:** Best RECIST response within 12 months of randomisation in the intention-to-treat population

		**Irinotecan group (n=230)**	**IrPan group (n=230)**
Complete response		0	3 (1%)
Partial response		27 (12%)	76 (33%)
Stable disease at 12 weeks		91 (40%)	56 (24%)
Progressive disease at 12 weeks		112 (49%)	95 (41)%)
	Radiological progression	69 (30%)	58 (25%)
	Clinical progression	10 (4%)	12 (5%)
	Death	27 (12%)	20 (9%)
	Inadequate data	6 (3%)	5 (2%)

Adverse events are summarised in table 3. Briefly, the toxicity of the two regimens was consistent with summaries of product characteristics for irinotecan and panitumumab, and in line with previous trials of anti-EGFR monoclonal antibodies. In terms of events that were grade 3 or higher, diarrhoea, lethargy, skin toxicity, infection, and neutropenia were all more common in the IrPan group than in the irinotecan group, as were any haematological, any non-haematological, or a grade 3 or higher toxicity of any type. However, there was no increase in the number of deaths attributed wholly or partly to treatment (three patients with irinotecan, two patients with IrPan), or in 60-day all-cause mortality (12 patients with irinotecan, 14 patients with IrPan).

**Table 3:** Adverse events

		Irinotecan group (n=218*)					IrPan group (n=219*)					**p value**†**grade ≥3 IrPan *vs* irinotecan**
		Grade 1	Grade 2	Grade 3	Grade 4	Grade 5	Grade 1	Grade 2	Grade 3	Grade 4	Grade 5	
Non-haematological												
	Nausea	91 (42%)	41 (19%)	13 (6%)	0	0	86 (39%)	40 (18%)	20 (9%)	0	0	0·21
	Vomiting	44 (20%)	36 (16%)	15 (7%)	0	0	46 (21%)	32 (15%)	13 (6%)	2 (1%)	0	0·99
	Diarrhoea (>24 h post-infusion)	86 (40%)	49 (22%)	38 (17%)	0	1 (<0·5%)	67 (31%)	64 (29%)	59 (27%)	4 (2%)	1 (<0·5%)	0·0053
	Diarrhoea (<24 h post-infusion)	17 (8%)	8 (4%)	3 (1%)	0	0	18 (8%)	16 (7%)	1 (<0·5%)	1 (<0·5%)	0	0·69
	Constipation	64 (29%)	17 (8%)	0	1 (<0·5%)	0	54 (25%)	23 (11%)	0	0	0	0·50
	Abdominal pain	61 (28%)	38 (17%)	12 (6%)	0	0	58 (26%)	23 (11%)	14 (6%)	0	0	0·69
	Skin toxicity	47 (22%)	8 (4%)	0	0	0	50 (23%)	103 (47%)	40 (18%)	1 (<0·5%)	0	<0·0001
	Nail toxicity	25 (11%)	0	0	..	..	51 (23%)	11 (5%)	5 (2%)	..	..	0·061
	Alopecia	43 (20%)	141 (65%)	..	..	..	70 (32%)	112 (51%)	..	..	..	N/A
	Lethargy	78 (36%)	78 (36%)	24 (11%)	0	0	67 (31%)	75 (34%)	43 (20%)	2 (1%)	0	0·0063
	Headache	26 (12%)	8 (4%)	0	0	0	24 (11%)	2 (1%)	0	0	0	N/A
	Dizziness	36 (17%)	4 (2%)	2 (1%)	0	0	35 (16%)	4 (2%)	2 (1%)	0	0	1·00
	Chills or non-neutropenic fever	33 (15%)	3 (1%)	5 (2%)	0	0	38 (17%)	13 (6%)	2 (1%)	2 (1%)	0	0·73
	Infection (including febrile neutropenia)	14 (6%)	19 (9%)	19 (9%)	1 (<0·5%)	2 (1%)	7 (3%)	26 (12%)	38 (17%)	4 (2%)	0	0·0072
	Any non-haematological	11 (5%)	126 (58%)	76 (35%)	2 (1%)	3 (1%)	4 (2%)	90 (41%)	112 (51%)	12 (5%)	1 (<0·5%)	<0·0001
Haematological‡												
	Neutropenia	19 (9%)	20 (9%)	8 (4%)	17 (8%)	2 (1%)	27 (12%)	15 (7%)	18 (8%)	30 (14%)	0	0·0082
	Thrombocytopenia	16 (7%)	1 (<0·5%)	0	0	0	26 (12%)	4 (2%)	4 (2%)	1 (<0·5%)	0	0·06
	Anaemia	91 (42%)	27 (12%)	2 (1%)	1 (<0·5%)	0	98 (45%)	22 (10%)	7 (3%)	1 (<0·5%)	0	0·13
	Any haematological	82 (38%)	37 (17%)	8 (4%)	18 (8%)	2 (1%)	88 (40%)	23 (11%)	18 (8%)	30 (14%)	0	0·012
Any of the above toxicities		9 (4%)	122 (56%)	64 (29%)	20 (9%)	3 (1%)	4 (2%)	83 (38%)	94 (43%)	37 (17%)	1 (<0·5%)§	<0·0001

Data are n (%), and are for toxicities reported within 12 weeks of randomisation at grade 3 or higher in more than 2% of patients.

* The population for adverse event reporting is patients who received at least one dose of the allocated treatment, and for whom at least one case record form was received to provide adverse event or serious adverse event data.

† Post-hoc univariate χ^2^ test (Fisher's exact test when number of events is five or fewer) of difference in proportion of grade 3–5 events, not adjusting for multiple testing.

‡ Nadir blood counts were not obtained routinely.

§ One treatment-related death (with neutropenic sepsis), was reported more than 12 weeks after randomisation. “..” is used where the grading does not exist under NCI CTCAE guidance. 0 is used where the grading does exist but no patients experienced it. IrPan=irinotecan plus panitumumab.

13 patients did not receive any trial treatment (seven allocated to irinotecan, six to IrPan). Of those who did, 66 (30%) of 223 patients on irinotecan and 89 (40%) of 224 patients on IrPan needed an irinotecan dose modification during cycles 1–4; panitumumab dose modifications were required for 60 (27%) of 224 patients during cycles 1–4.

Of patients who were alive and completed quality-of-life questionnaires at 24 weeks post-randomisation (111 [70%] of 158 patients in the irinotecan group, 125 [75%] of 167 patients in the IrPan group), EORTC QLQ-C30 global quality-of-life scores at 24 weeks, after adjusting for baseline quality of life, were moderately better^14^ with IrPan than with irinotecan alone (mean 56·4 *vs* 49·5; difference 7·0, 0·6–13·4, two-sided p=0·032). By contrast with the global scores, and in keeping with the clinician-reported adverse events (table 3), quality-of-life symptom scores were worse with IrPan (data not shown).

Of the 460 patients in the primary population, 137 (30%) were classified as having any mutation and 323 (70%) were all wild type (table 1 and figure 2). Mutations affecting more than one gene were uncommon (figure 2).

Mutation status was first assessed as a prognostic variable for overall survival in patients treated with irinotecan alone (table 4). When corrected for prognostic variables (minimisation factors), patients in the any mutation group had inferior survival to all wild-type patients (p=0·049). Patients with *BRAF*-mutated tumours had worse overall survival than did all-wild-type patients (table 4). However, the numbers of patients in these subgroup analyses is small and these exploratory results should be interpreted with caution.

**Table 4:** Prognostic analysis

	**Total (n)**	**Irinotecan (n)**	**Hazard ratio (95% CI) for overall survival comparison *vs* wild-type in irinotecan group**
All wild-type	323	163	NA
Any mutation	137	67	1·36 (1·00–1·83); p=0·049
*BRAF* mutation	68	31	1·56 (1·03–2·37); p=0·035
*NRAS* mutation	29	10	1·15 (0·60–2·21); p=0·67
*KRAS*_c.146_ mutation	17	8	1·77 (0·85–3·69); p=0·13
*PIK3CA* mutation	32	21	1·11 (0·68–1·80); p=0·69

A hazard ratio greater than one indicates worse survival for patients in mutation group compared with all wild-type patients.

**Figure 4:**
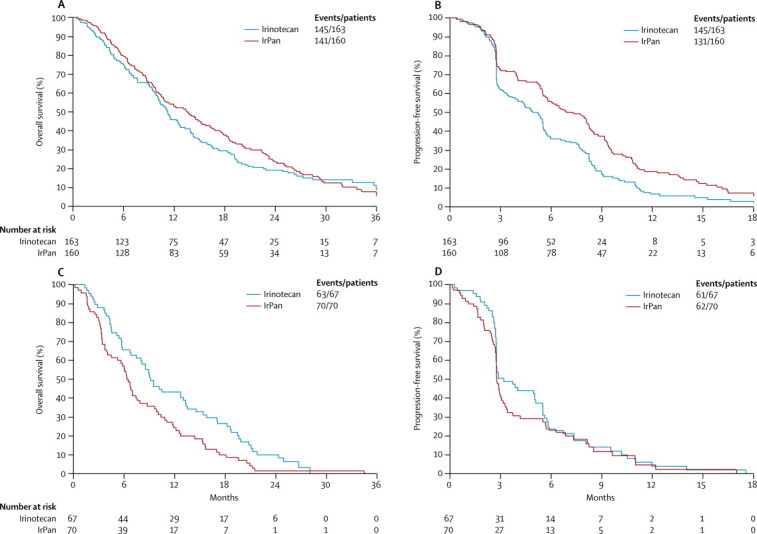
Key efficacy endpoints, by mutation status (A) Overall survival in patients with no mutations. (B) Progression-free survival in patients with no mutations. (C) Overall survival in patients with any mutation. (D) Progression-free survival in patients with any mutation. IrPan=irinotecan plus panitumumab.

**Figure 5:**
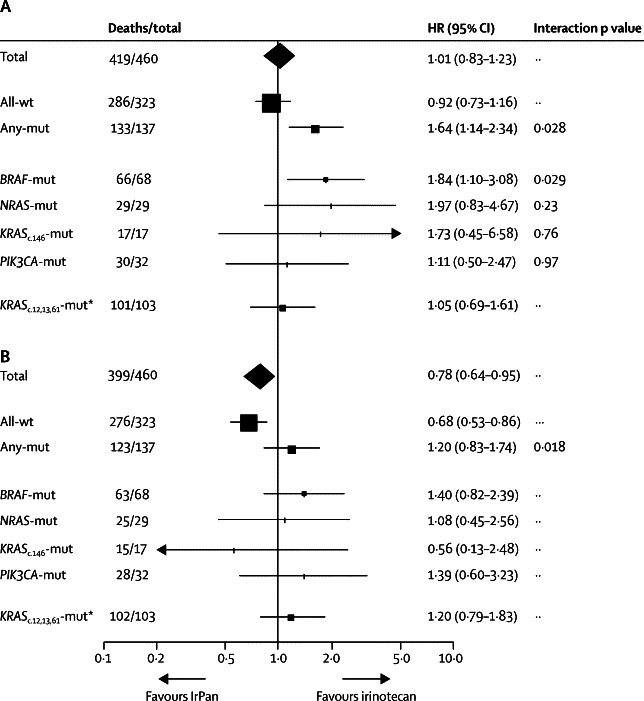
Subgroup analysis, by mutation status Forest plot of (A) overall survival and (B) progression-free survival. Hazard ratios (HRs) and 95% CIs are corrected for minimisation factors, comparing irinotecan plus panitumumab (IrPan) *vs* irinotecan alone. All-wt=no mutations detected. Any-mut=any mutation detected. *Patients randomised before the protocol amendment in June 10, 2008, and genotyped retrospectively.

We then assessed mutation status as a predictive biomarker of the effect of panitumumab treatment on overall survival, PFS, and response rate, using tests of interaction with mutation status (any mutation *vs* all wild-type), corrected for prognostic variables. The interaction tests assess whether there is a true difference between the two subpopulation in the impact of adding panitumumab to irinotecan. The interaction test was positive for all three outcome measures: overall survival (p=0·028, Figure 4, Figure 5), progression-free survival (p=0·018, figure 5B) and response rate (p=0·0095, appendix). In patients with all-wild-type tumours, those in the IrPan group had better PFS and response rate than did those in the irinotecan groups (Figure 4, Figure 5, appendix), but we detected no between-group difference in terms of overall survival (figure 5). By contrast with this finding, in patients with any mutation, panitumumab had no effect on PFS or response rate (figure 5B, and appendix) and an adverse effect on overall survival (Figure 4, Figure 5). For individual mutations, the small numbers provide insufficient power to confidently detect or refute interactions between treatment effect and mutation status, so results are exploratory. For patients with *BRAF*-mutated tumours, there was a suggestion of harm with panitumumab (figure 5A). The effect of panitumumab on PFS and response rate in the individual mutation subgroups gave less consistent results than for overall survival (figure 5B, appendix). A breakdown of PFS events (by all wild-type *vs* any mutation) is shown in the appendix.

A sensitivity analysis including only patients with a full set of data in the all-wild-type group gave similar HRs and effect sizes to those for the whole all-wild-type population for all endpoints (appendix). Similarly a sensitivity analysis excluding individuals with *PIK3CA* mutations did not alter the findings (appendix). Separate analysis of *PIK3CA* exon 9 and 20 is of minimal relevance given the small numbers in these groups (appendix).

In view of the disparity between treatment effects on overall survival and PFS, we did a post-hoc analysis of survival after progression. Post-progression survival was reduced in patients in the IrPan group, and this difference was more pronounced in the any-mutation population (appendix).

494 patients were enrolled and randomly allocated before the protocol modification, 329 to irinotecan or irinotecan plus panitumumab. Of these, 261 had tumour samples available for retrospective analysis (132 in the irinotecan group and 129 in the IrPan group). 103 (39%; 53 in the irinotecan group, 50 in the IrPan) had a *KRAS*_c.12,13,61_ mutation (including 17 patients with Gly13Asp mutations; ten in the irinotecan group, seven in the IrPan group). We detected no treatment effect (beneficial or detrimental) with panitumumab in these individuals (figure 5 and appendix). We detected no benefit of panitumumab in the Gly13Asp mutation subgroup (data not shown).

## Discussion

In our trial, the addition of panitumumab to irinotecan for patients with *KRAS* wild-type tumours had no effect on overall survival, which was our primary endpoint. However, the addition of panitumumab improved the secondary outcome measures of PFS and the proportion of patients who had a response. Our findings are in keeping with the emerging pattern of clinical effect of anti-EGFR monoclonal antibody therapy in patients with *KRAS* wild-type colorectal cancer (panel).

Panel **Research in context**

**Systematic review**

We searched Medline using OvidSP for published randomised clinical trials in advanced colorectal cancer involving an anti-EGFR monoclonal antibody. We used the following searchterms: “colorectal”, “randomis[z]ed”, “panitumumab”, “cetuximab”. Our last search was done on Jan 22, 2013, and we used no language restrictions. We identified 12 trials that included randomisation to standard treatment plus or minus anti-EGFR monoclonal antibodies.^1^, ^2^, ^3^, ^17^, ^18^, ^23^, ^24^, ^25^, ^26^, ^27^, ^28^, ^29^ In none was *KRAS* status determined before randomisation, but for ten trials results have been published by *KRAS* status (usually confined to codons 12–13) either within the primary analysis or as a secondary report. Two useful meta-analyses of these ten trials have been done, drawing attention to a lack of consistency in outcomes, especially among patients with *KRAS* wild-type tumours.^16^, ^30^ Unexplained antagonistic interactions with other cancer drugs have been proposed: combinations with bevacizumab, capecitabine, or oxaliplatin have produced poor results, whereas single-agent therapy or combinations with irinotecan or fluorouracil have had more success. Another trend, also unexplained, is toward worsening outcomes with earlier stage disease: clear benefit in the third-line setting, lesser benefit in second line, mixed results in first-line, and negative results in two large surgical adjuvant trials.^31^, ^32^

**Interpretation**

Our findings for *KRAS*_c.12,13,61_ wild-type patients show that prospective molecular stratification is feasible and gives outcomes consistent with these previous retrospective analyses. As in the two previous second-line studies of panitumumab,^24^, ^25^ we saw improved response rate and progression-free survival, but with no effect on overall survival. However, findings from other trials of alternative novel agents should also be considered. For example, randomised trials have shown small, but statistically significant, improvements in survival when either bevacizumab^33^ or aflibercept^34^ is added to chemotherapy in the second-line setting. Thus, only if further refinement of molecular selection resulted in a substantial survival benefit from therapeutic antibodies targeting EGFR would they become the preferred option in this clinical setting.

To the best of our knowledge, PICCOLO is the first randomised trial in advanced colorectal cancer to have introduced prospective testing of mutation status to determine patients' randomisation and treatment. It shows that rapid testing by a central laboratory is feasible in a multi-centre research setting. In addition to prospective stratification by *KRAS*_c.12,13,61_ status, PICCOLO included a prospectively planned, retrospective analysis of other candidate mutations in the MEK/AKT activation pathway. Determination of the effect of less common mutations is challenging, because any randomised trial powered for a common group (eg, *KRAS* wild-type) is inevitably underpowered to detect or exclude potentially clinically important effects in rarer subgroups (eg, *BRAF* mutation). In PICCOLO, we grouped several candidate mutations in the EGFR signalling pathway, allowing a higher-powered comparison of any mutation versus all-wild-type than would be possible for individual mutations. This approach does not mean that every mutation selected is individually important, nor that the list is exhaustive; it does, however, provide evidence that interactions exist. The choice of mutations was based on their roles as oncogenes in EGFR signal transduction, coupled with data from grouped retrospective analyses of non-randomised patients suggesting clinical relevance.^15^, ^16^ Least certain is the relevance of *PIK3CA*, where non-randomised data has implicated exon 20, but not exon 9, as a negative biomarker.^15^ The small number of patients with mutations at *PIK3CA* in PICCOLO precludes firm conclusions; however, patients with mutations at exon 9 did not benefit from panitumumab, and the sensitivity analysis excluding *PIK3CA* from the list did not alter that finding (appendix).

An inconsistent, but nonetheless worrying, finding in trials of anti-EGFR monoclonal antibodies is that patients who do not benefit from treatment are potentially harmed. Findings of a meta-analysis including ten randomised controlled trials in advanced colorectal cancer showed, although not statistically significant, a trend towards worse PFS in patients with *KRAS* mutations (HR 1·11, 95% CI 0·97–1·27);^17^ three of the ten trials showed a statistically significant detrimental effect.^3^, ^18^, ^19^ Drug-specific adverse interactions with oxaliplatin and bevacizumab have been inferred, although on no basis and with no mechanism proposed, and this has led to a supposition that anti-EGFR monoclonal antibodies are better paired with irinotecan than with other drugs.^16^

We have now shown in this prospective randomised trial, including irinotecan, but neither oxaliplatin nor bevacizumab, that the *KRAS* wild-type population contains subpopulations for whom anti-EGFR monoclonal antibodies are similarly detrimental. The all wild-type population of patients benefited from panitumumab, with a high response rate (70 [44%] of 160 patients) and improved PFS (HR 0·68; 95% CI 0·53–0·86); but we saw no statistically significant difference in overall survival between the two groups (Figure 4, Figure 5). By contrast with these findings, in patients with any mutation, we detected a potential detrimental effect of panitumumab in terms of PFS and of overall survival (Figure 4, Figure 5).

This disparity between effects on PFS and overall survival is substantiated by our findings that suggested shorter survival after progression following irinotecan and panitumumab, especially in the any-mutation population (appendix). Several possible explanations must be considered. Imbalanced post-trial treatment with more effective salvage of patients in the control group is unlikely to have been a major factor: the use of anti-EGFR monoclonal antibodies was carefully monitored, but these drugs were not funded in the UK at the time of the trial and were received by only 13 (6%) patients in the control group in the 3 months after progression. Although the fact that full data were not collected for other salvage treatments is a weakness of this study, there is no reason to believe that these would have been imbalanced. Ascertainment bias—a lower threshold for diagnosing progression in patients in the control group—is also unlikely, because there was a higher rate of confirmed radiological progression in the control group than in the experimental group (appendix). The third explanation is that panitumumab caused accelerated tumour growth during or after therapy. This seems to have been the case in the population of patients with any mutations, in which the progression event was death for a higher proportion, and where substantially inferior survival after progression suggests more rapid tumour growth after stopping treatment (appendix).

The demonstration of detriment within subpopulations of *KRAS* wild-type patients casts doubt on the current select-out approach to anti-EGFR monoclonal antibody therapy, in which the default position is to treat unless the patient is in a group of well-proven inefficacy (eg, with a mutation at *KRAS*_c.12,13_). Urgent clarification of subpopulations at risk of harm is important, but positive biomarkers are also needed, to allow a change to a select-in strategy, using anti-EGFR monoclonal antibodies in only well-defined molecular groups with proven efficacy. Potential, although not validated, positive biomarkers include EGFR ligands^20^ and EGFR copy number.^21^, ^22^

For the individual mutations tested in PICCOLO, the numbers of patients were insufficient to provide clear results. The exception was in patients with mutations in *BRAF*, the most common mutation, in whom we detected a detrimental effect of panitumumab on overall survival (HR 1·84, 95% CI 1·10–3·08). Findings from previous studies of patients with *BRAF* mutations are inconsistent. Large, but non-randomised, series suggest that anti-EGFR monoclonal antibodies are inactive in *BRAF*-mutated cancers;^14^, ^15^, ^17^ however, retrospective analysis of *BRAF* status in two randomised trials, although showing a low response rate in patients with *BRAF* mutations, showed no evidence of a negative interaction on PFS.^2^

The data presented here substantiate the activity of anti-EGFR monoclonal antibodies in advanced colorectal cancer, but also show the need for selection strategies beyond the current reliance on *KRAS*. Rapid independent validation or refutation of the PICCOLO findings is feasible using existing clinical trial biobanks. Urgent refinement of both negative and positive selection biomarkers using preclinical studies and both retrospective and prospective clinical trial analysis are needed if best use is to be made of an effective targeted therapy for the benefit of patients.

For the **study protocol** see http://ctru.leeds.ac.uk/Piccolo
