# Supplementary material for: Panitumumab and irinotecan versus irinotecan alone for patients with KRAS wild-type, fluorouracil-resistant advanced colorectal cancer (PICCOLO): a prospectively stratified randomised trial
Source: Lancet Oncol. 2013 Jul;14(8):749–59. doi: 10.1016/S1470-2045(13)70163-3 (PMC3699713; doi:10.1016/S1470-2045(13)70163-3)
Supplement: Supplementary appendix [file mmc1.pdf]

THE LANCET Oncology

Supplementary appendix

This appendix formed part of the original submission and has been peer reviewed. We post it as supplied by the authors.

Supplement to: Seymour MT, Brown SR, Middleton G, et al. Panitumumab and irinotecan versus irinotecan alone for patients with *KRAS* wild-type, fluorouracil-resistant advanced colorectal cancer (PICCOLO): a prospectively stratified randomised trial. *Lancet Oncol* 2013; published online May 29. http://dx.doi.org/10.1016/S1470-2045(13)70163-3.

Appendix to Panitumumab and irinotecan versus irinotecan alone for patients with *KRAS* wild-type, fluorouracil-resistant advanced colorectal cancer (PICCOLO): a prospectively stratified randomised trial

1. Participating centres and principal investigators
2. Laboratory methods
3. Additional clinical data
4. Sensitivity analyses

1) Participating centres and Principal Investigators

Addenbrookes, Cambridge (PI: Charles Wilson); Airedale, Keighley, (PI Michael Crawford); Bradford Royal Infirmary (PI: Andy Conn); Bristol (PI: Stephen Falk); Huddersfield & Halifax (PI: Jo Dent); Charing Cross & Hammersmith, London (PI: Charles Lowdell); Cheltenham & Gloucester (PI: Kim Benstead); Clatterbridge (PI: Sun Myint); Dorset County (PI: Richard Osborne); Eastbourne (PI: Fiona McKinna); Edinburgh Cancer Centre, Western General, Edinburgh (PI: Lesley Dawson); Grimsby (PI: Rajarshi Roy); Hereford County (PI: Nick Reed); Hinchingbrooke, Cambridge (PI :Li Tee Tan); James Cook, Middlesbrough (PI: Nicholas Wadd); Kent and Canterbury Hospitals (PI: Catherine Harper-Wynne); Maidstone (PI: Mark Hill); Mount Vernon Hospital, Middlesex (PI: Rob Glynne-Jones); New Cross Hospital (PI: Mark Churn); Northampton (PI: Craig Macmillan); Nottingham City (PI: Dr Vanessa Potter); Peterborough (PI: Karen McAdam); Poole & Bournemouth (PI: Tamas Hickish); Burton (PI: Prabir Chakraborti); Devon & Exeter (PI: Melanie Osborne); Royal Free Hospital, London (PI: Astrid Mayer); Royal Hampshire (PI: Dr Nolan); Royal Marsden (PI: Ian Chau); Scunthorpe (PI: Abdel Hamid); Swansea (PI:John Wagstaff); Leeds (PI Matt Seymour); South Tyneside (PI: Ashraf Azzabi); Bart's London (PI: Sarah Slater); St Luke's Guildford (PI: Gary Middleton); St Mary's, London (PI: Susan Cleator); St Mary's/QA Portsmouth (PI: Ann O'Callaghan); St Thomas's/QE London (PI: Nick Maisey); Stafford (PI: Selvaraj Giridharan); Swindon (PI: Claire Blesing); Torbay (PI: Nangi Lo); UCL/N.Middx/Harlow (PI: John Bridgewater); Coventry & Warwickshire (PI: Robert Grieve); Velindre, Cardiff (PI: Tim Maughan); Wansbeck, Northumberland (PI: Werner Dobrowsky); West Middlesex (PI: Pippa Riddle); Weston Park, Sheffield (PI: Jonathan Wadsley); Worthing/W.Sussex/Royal Sussex (PI: Andrew Webb); Yeovil, Somerset (PI: Clare Barlow); Ysbyty Gwynedd, Bangor (PI: Catherine Bale); Ysbyty Maelor & Glan Clwyd (PI: Simon Gollins);

2) Laboratory Methods

Formalin-fixed, paraffin-embedded tumor tissue was retrieved, anonymized and encoded with the patient's trial number at the treating hospital, then sent to the research laboratory. Here, all staff were blind to the patients' treatment allocation and clinical outcomes. The laboratory processing and storage of samples was under Ethical approval, and the laboratory adheres where possible to GCLP guidelines and participates annually in the UK National External Quality Assessment Service (NEQAS) scheme.

Areas containing the highest density of tumour cells were identified on a hematoxylin and eosin stained section. Six to nine 5 µm sections were used per extraction, depending upon the tumour area per slide. The sections were macrodissected with a scalpel blade, to ensure that only the tumour-rich areas were used. DNA was extracted using the Qiagen QiaAmp Micro Kit (Qiagen, Crawley, UK) following the manufacturer's instructions. The final DNA was eluted into 25 microlitres of laboratory-grade water and the concentration determined using a Nanodrop ND-1000 spectrophotometer (Labtech International, Uckfield, East Sussex, UK). The DNA was stored at $4^{o}C$ until required.

Primers for PCR amplification and Pyrosequencing analysis were designed using proprietary Pyrosequencing assay design software version 2.0.1.15 (Qiagen, Crawley, UK). PCR reactions contained 12.5µl of Qiagen HotStarTaq Master Mix (Qiagen, Crawley, UK), additional magnesium chloride to a final concentration of 2mM, 200nM each of forward and reverse primers, 20ng of tumour DNA and sufficient water to make a final volume of 25µl. Thermal cycling conditions for all amplicons were 94°C for 12 minutes followed by 40 cycles of 94°C for 10 seconds, 55°C for 20 seconds and 72°C for 20 seconds. PCR products were sequenced by Pyrosequencing on a PyroMark ID system (Qiagen, Crawley, UK) following the manufacturer's protocols. Data was analysed by visual inspection of Pyrograms and by statistical analysis of peak height data.

Table 1 (appendix): PCR and Pyrosequencing primer sequences for amplification and analysis of *KRAS* codons 12 and 13, *KRAS* codon 61, *KRAS* codon 146, *NRAS* codons 12 and 13, *NRAS* codon 61, *PIK3CA* codon 542, *PIK3CA* codons 545 and 546, *PIK3CA* codon 1047 and *BRAF* codon 600.

Region of interest	PCR primers (5' → 3')	Pyrosequencing primer (5' → 3')	PCR amplicon length (bp)
KRAS codons 12 and 13	Fwd: GGCCTGCTGAAAATGACTGA<br>Rev: biotin-AGCTGTATCGTCAAGGCACTCT	AAACTTGTGGTAGTTGGA	80
KRAS codon 61	Fwd: AATTGATGGAGAAACCTGTCTCTT<br>Rev: biotin- TCCTCATGTACTGGTCCCTCATT	GGATATTCTCGACACAGC	86
KRAS codon 146	Fwd: TCAGGACTTAGCAAGAAGTTATGG<br>Rev: biotin- TGCAGAAAACAGATCTGTATTTAT	GTGTTACTTACCTGTCTTGT	100
NRAS codons 12 and 13	Fwd: CTTGCTGGTGTGAAATGACTGAG<br>Rev: biotin-TGGATTGTCAGTGCGCTTTTC	CTGGTGGTGGTTGGA	79
NRAS codon 61	Fwd: biotin-GAAACCTGTTTGTTGGACATACTG<br>Rev: TCGCCTGTCCTCATGTATTG	CTCTCATGGCACTGTACT	83
PIK3CA codon 542	Fwd: biotin-AAAGCAATTTCTACACGAGATCC<br>Rev: GCACTTACCTGTGACTCCATAGA	TTCTCCTGCTCAGTGAT	79
PIK3CA codons 545 and 546	Fwd: ACAGCTCAAAGCAATTTCTACACG<br>Rev: biotin- TCCATTTTAGCACTTACCTGTGAC	GATCCTCTCTCTGAAATC	95
PIK3CA codon 1047	Fwd: biotin-TGAGCAAGAGGCTTTGGAGTAT<br>Rev: TGCTGTTTAATTGTGTGGAAGATC	GTTGTCCAGCCACCA	102
BRAF codon 600	Fwd: TGAAGACCTCACAGTAAAAATAGG<br>Rev: biotin-TCCAGACAACTGTTCAAACTGAT	TGATTTTGGTCTAGCTACA	91

3) Additional clinical data

Table 2 (appendix): Reasons for stopping treatment and post-trial EGFR-mAb therapy

	Irinotecan<br>n	IrPan<br>n
***KRAS*$_{c.12,13,61}$-wt**	**230**	**230**
**Patients stopped treatment**	220	207
**Reason for stopping**		
Progression	122 (55.5)	110 (53.1)
Death	34 (15.5)	36 (17.4)
Toxicity	12 (5.5)	20 (9.7)
Patient choice / unknown	52 (23.6)	41 (19.8)
**Post-trial EGFR-mAb therapy received**	14 (6.4)	1 (0.5)

Table 3 (appendix): Breakdown of progression-free survival events

**Progression event**	**Irinotecan<br>n (%)**	**IrPan<br>n (%)**
***KRAS*$_{c.12,13,61}$-wt**	**206**	**193**
Radiological progression	147 (71.4)	128 (66.3)
Clinical progression	10 (4.9)	12 (6.2)
Death	49 (23.8)	53 (27.5)
**All-wt**	**145**	**131**
Radiological progression	103 (71.0)	94 (71.8)
Clinical progression	5 (3.4)	5 (3.8)
Death	37 (25.5)	32 (24.4)
**Any-mut**	**61**	**62**
Radiological progression	44 (72.1)	34 (54.8)
Clinical progression	5 (8.2)	7 (11.3)
Death	12 (19.7)	21 (33.9)

Figure 1 (appendix): Subgroup analysis by mutation status: Response Rate

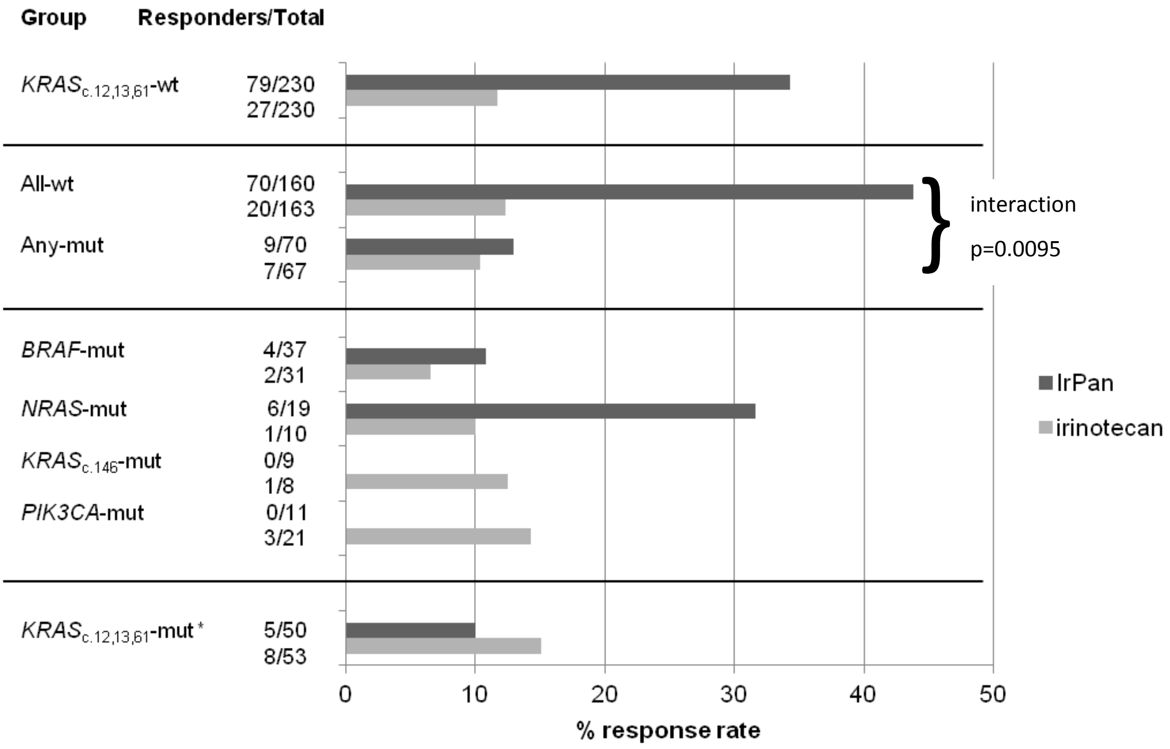

RECIST response rate by treatment arm in patients in the primary analysis population. "All-wt" = no mutations detected; "Any-mut" = any mutation detected.

*$KRAS_{12,13,61}$-mut: patients randomised prior to the protocol amendment in June 2008 and genotyped retrospectively (see main paper Figures 1 and 2)

Figure 2 (appendix): Survival after progression

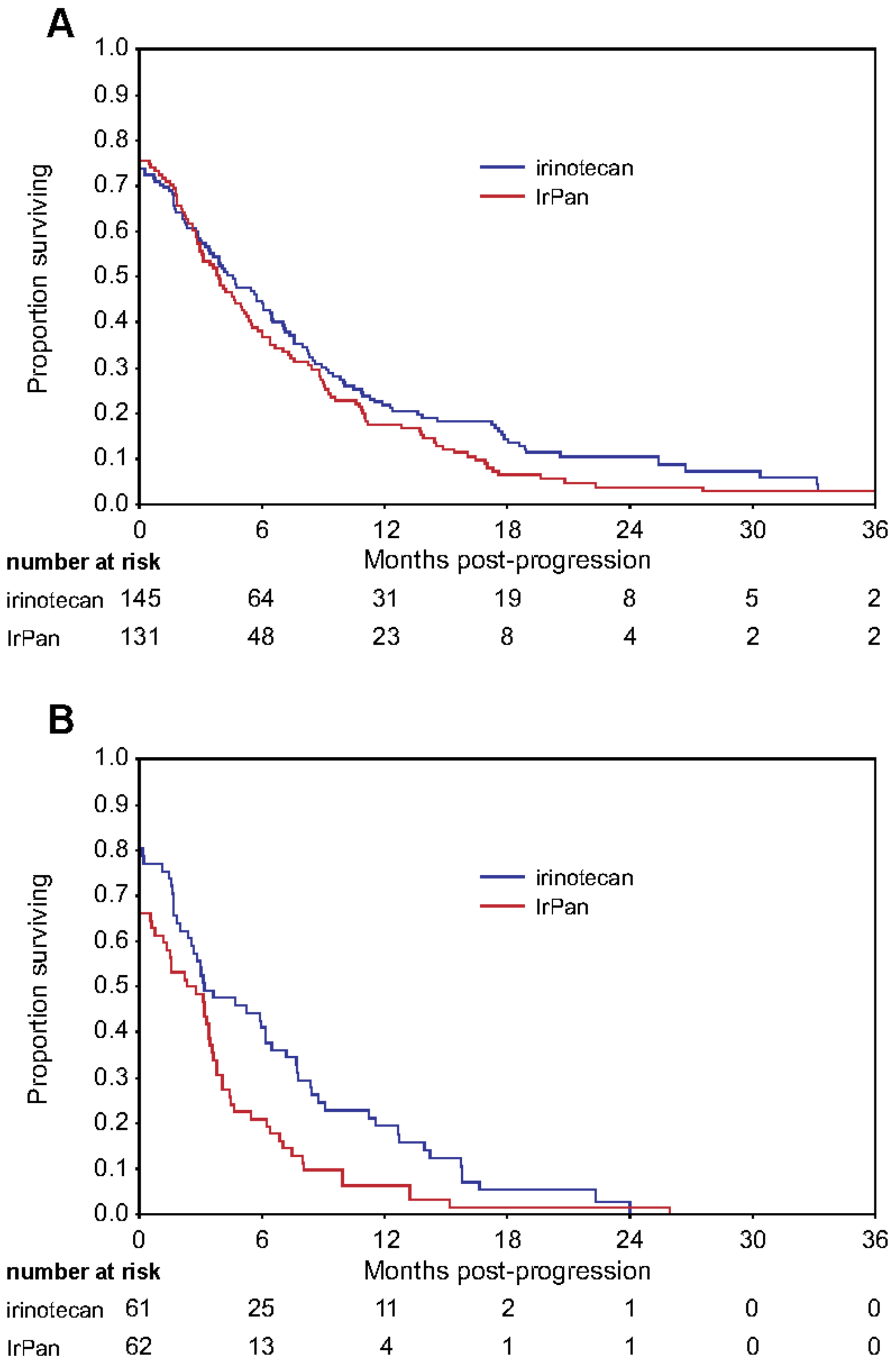

(A) All-wt patients (B) Any-mut patients. Patients whose progression event was death are classed as an event at time zero. Please view this figure alongside the PFS and OS figures shown in the main paper, Figure 5A-D

4) Sensitivity analyses

a) All-mut status definition sensitivity analysis

In the main analysis, patients with an incomplete set of genetic data are included in the "All-wt" group provided their status is wt at every codon for which data is available. In Table 4 (Webappendix), these data are shown in the top row, and for comparison, in the second row, the data for patients with a full set of data confirming wild-type status at all 12 loci under investigation (n=237). Data are given for each efficacy endpoint for this patient group and the tests for interaction with "any-mut" status.

Table 4 (appendix): Sensitivity analysis for missing mutation status

	OS	PFS	RECIST Response	
	Hazard ratio* (95% CI)	Hazard ratio* (95% CI)	Irinotecan	IrPan
**All-wt as defined in main analysis (no mutations detected, n=323)**	0.92 (0.73, 1.16)	0.68 (0.53, 0.86)	20/163 (12.3%)	70/160 (43.8%)
**Confirmed wt at every locus (n=237)**	0.98 (0.74, 1.29)	0.61 (0.46, 0.82)	17/124 (13.7%)	48/113 (42.5%)
**Any-mut (n=137)**	1.64 (1.14, 2.34)	1.20 (0.83, 1.74)	7/67 (10.4%)	9/70 (12.9%)
**Interaction p-value (any-mut vs. confirmed wt at every locus)**	*0.071*	*0.012*	*0.025*	

* For IrPan vs. irinotecan. A hazard ratio <1 indicates improved survival with IrPan compared to irinotecan alone

b) PIK3CA sensitivity analysis

In the main analysis, mutations at *PIK3CA* exon 9 or 20 are grouped along with mutations in the *RAS-RAF-MEK* pathway for inclusion in the "Any-mut" group, shown here in the second row (n=137) of Table 5 (Webappendix). However the role of *PIK3CA* in EGFR signalling is less direct. The sensitivity analysis presented here shows treatment impact when the 26 patients with only a *PIK3CA* mutation are not included (third row, n=111). Also shown are the treatment effects in patients with only a *PIK3CA* mutation, although numbers here are extremely small, and the confidence intervals correspondingly wide.

Table 5 (appendix): Sensitivity analysis for PIK3CA mutations

	OS	PFS	RECIST Response	
	Hazard ratio* (95% CI)	Hazard ratio* (95% CI)	Irinotecan	IrPan
**All-wt (n=323)**	0.92 (0.73, 1.16)	0.68 (0.53, 0.86)	20/163 (12.3%)	70/160 (43.8%)
**Any-mut as defined in main analysis (any mutation including *PIK3CA*, n=137)**	1.64 (1.14, 2.34)	1.20 (0.83, 1.74)	7/67(10.4%)	9/70 (12.9%)
**Any mutation in *BRAF*, *NRAS* or $KRAS_{c.146}$ (n=111)**	1.75 (1.17, 2.61)	1.16 (0.77, 1.75)	4/49 (8.2%)	9/62 (14.5%)
***PIK3CA* exon 9 mutation: (n=19)**	1.49 (0.43, 5.17)	1.80 (0.57, 5.67)	3/13 (23.1%)	0/6 (0%)
***PIK3CA* exon 20 mutation: (n=7)**	0.77 (0.14, 4.30)	0.42 (0.05, 3.80)	0/5 (0%)	0/2 (0%)
